# Treatment of postmenopausal osteoporosis with recombinant human parathyroid hormone and electromagnetic field

**DOI:** 10.1007/s40520-025-02932-w

**Published:** 2025-02-22

**Authors:** Miao Xuan, Bo Wang, Wanrong Bi, Ying Li, Lige Song, Zhuangli Xie, Qi Liu, Xiuzhen Zhang

**Affiliations:** 1 Department of Endocrinology,School of Medicine，Tongji Hospital, Tongji University, Shanghai, 200065 China; 2 Department of Endocrinology, School of Medicine, Yangpu Hospital, Tongji University, Shanghai, 200090 China; 3 School of Medicine, Tongji University, Shanghai, 200331 China

**Keywords:** Electromagnetic field, Postmenopausal osteoporosis, Bone density, Recombinant human parathyroid hormone

## Abstract

**Objective:**

This study aimed to investigate the effect of electromagnetic field (EMF) combined with recombinant human parathyroid hormone (rhPTH) on bone mineral density (BMD) and bone turnover indicators in postmenopausal osteoporosis (PMOP) patients.

**Methods:**

A total of 336 PMOP patients were randomly assigned into three groups: EMF + rhPTH group (*n* = 115), rhPTHx group (*n* = 113) and EMF group (*n* = 108). The lumbar spine and femoral neck BMDs were measured before treatment and at 6, 12, and 18 months after treatment. Blood calcium, bone alkaline phosphatase (BSAP), type I procollagen N-terminal peptide (PINP), and type I collagen C-terminal peptide/creatinine ratio (CTX/Cr) levels were measured before treatment and at 3, 6, 12, and 18 months after treatment.

**Results:**

The lumbar spine BMD was significantly increased at 6, 12, and 18 months after treatment, and the neck BMD was increased markedly at 18 months in both EMF + rhPTH group and rhPTH group as compared to those before treatment. There was significant difference in the lumbar spine BMD between EMF + rhPTH group and EMF group and between rhPTH group and EMF group at 6, 12, and 18 months after treatment. In the EMF + rhPTH group, at 3, 6, 12, and 18 months after treatment, blood calcium level was increased by 5.2%, 2.8%, 2.7%, and 3.1%, respectively; BASP level was increased by 80.9%, 120.3%, 84.1%, and 67.7%, respectively; PINP level was increased by 65.4%, 79.7%, 89.7%, and 74.5%, respectively; CTX/Cr was increased by 80.9%, 120.3%, 84.1%, and 67.7%, respectively; the bone metabolism indicators were markedly higher than those before treatment. In the rhPTH group, at 3, 6, 12, and 18 months after treatment, blood calcium level was increased by 5.1%, 3.3%, 3.0%, and 2.1%, respectively; BSAP level was increased by 51.6%, 81.4%, 101.1% and 56.3% respectively; PINP level was increased by 48.5%, 69.8%, 80.7% and 70.5% respectively; CTX/Cr was increased by 29.8%, 29.9%, 55.7%, and 44.8% respectively; the bone turnover indicators were significantly different from those before treatment (*P* < 0.01).

**Conclusion:**

The combination of EMF and rhPTH can significantly improve the bone turnover and BMD of PMOP patients, and may serve as a clinical treatment of PMOP.

## Introduction

Osteoporosis (OP) is a systemic disease characterized by reduced bone mass, degeneration of bone microstructure, increased bone fragility, and susceptibility to fractures [1]. Fractures caused by OP can lead to a decrease or loss of self-care of patients, even resulting in death, which imposes a heavy economic burden on the society and families. The postmenopausal osteoporosis (PMOP) has a relatively high prevalence, and may progress into severe OP or even cause fracture if it is not managed and treated in a timely manner [2]. Currently, the treatments of PMOP are generally based on supplementation of calcium and vitamin D, with the addition of drugs that can inhibit bone resorption and/or promote osteogenesis, but these treatments have some side effects and the therapeutic efficacy is still poor. Pulsed Electromagnetic Field (PEMF) is a non-invasive treatment used to promote bone healing and improve bone density. To date, few studies have conducted to investigate the physical therapy of PMOP with PEMF or extracorporeal shock wave [1]. Although treatment of OP with recombinant human parathyroid hormone (rhPTH) or PEMF has been reported [3], the therapeutic efficacy of rhPTH combined with PEMF has not been systemically investigated. This study aimed to investigate the therapeutic effects of PEMF and rhPTH on the bone mineral density (BMD) and bone turnover indicators in PMOP patients. Our findings may provide evidence on the clinical treatment of PMOP with rhPTH and PEMF.

## Subjects and methods

### Subjects

A total of 336 inpatients and outpatients with PMOP were recruited from the Department of Endocrinology, Tongji Hospital of Tongji University between December 2020 and December 2022. They were randomly assigned into three groups: PEMF and rhPTH group (Group A) (*n* = 115), rhPTH group (Group B) (*n* = 113) and PEMF group (Group C) (*n* = 108). The inclusion criteria were as follows: (1) OP was diagnosed as follows: lumbar spine and femoral neck BMDs were below 2.5 standard deviations of mean peak BMD of the same gender (T ≤ -2.5) or T value was <-1 and there was at least one definite feature of osteoporotic fragility fracture on imaging examinations; (2) Subjects did not take glucocorticoids or anti-osteoporosis drugs, and receive home replacement therapy (HRT) within prior one year; (3) Primary diseases of the liver (alanine aminotransferase > 100 U/L), kidney (serum creatinine concentration higher than 110 mmol/L), pituitary gland, thyroid gland, parathyroid gland, adrenal gland, gonads and blood system (such as multiple myeloma, leukemia, lymphoma, and myelodysplastic syndrome) that may affect bone turnover were excluded. Connective tissue diseases, osteogenesis imperfecta, bone tumors, and deformans osteitis were also excluded. The baseline characteristics are shown in Table 1. This study was approved by the Ethics Committee of Tongji Hospital, and all participants signed informed consent forms before study.

**Table 1:** Baseline clinical characteristics of patients in different groups

Parameters	Group A (*n* = 115)	Group B (*n* = 113)	Group C (*n* = 108)	F /χ^2^	*P*
Age (yr)	65.04 ± 7.10	65.23 ± 7.07	65.37 ± 7.38	0.058	0.943
Height (cm)	155.78 ± 5.15	153.98 ± 6.00	155.33 ± 5.12	1.727	0.179
Body weight (kg)	55.81 ± 9.92	57.07 ± 9.35	55.74 ± 9.09	0.696	0.499
BMI (kg/m^2^)	23.38 ± 4.48	24.14 ± 4.21	23.18 ± 4.08	1.597	0.204
Age of menopause (yr)	20.18 ± 9.48	20.22 ± 9.39	21.31 ± 9.88	0.487	0.615
Smoking (n)	2	1	1	0.448	0.799
Drinking (n)	3	3	2	0.192	0.908
BMD					
L1-4 (g/cm^2^)	0.782 ± 0.107	0.786 ± 0.101	0.785 ± 0.102	0.032	0.968
Neck (g/cm^2^)	0.657 ± 0.106	0.641 ± 0.109	0.657 ± 0.107	0.884	0.414
Bone turnover indicators					
PINP (pg/L)	70.03 ± 7.44	71.25 ± 7.85	69.97 ± 7.78	0.983	0.375
BSAP (µg/L)	20.55 ± 9.93	22.49 ± 9.14	21.03 ± 10.98	1.150	0.318
CTX/Cr (µg/mmol)	194.94 ± 50.80	194.23 ± 52.09	186.91 ± 57.71	0.759	0.469
Blood Ca (mmol/L)	2.34 ± 0.13	2.35 ± 0.15	2.36 ± 0.16	0.890	0.412
Blood P (mmol/L)	1.25 ± 0.15	1.25 ± 0.16	1.25 ± 0.14	0.004	0.996

## Methods

Group A: Patients received treatment with PEMF and rhPTH. The electromagnetic field therapy instrument (Beijing Yuhua International Technology Cooperation Co., Ltd; BG100A) was used for PEMF. First, patients received PEMF treatment for 5 months and then the PEMF treatment was initiated 1 month later. PEMF treatment lasted for 18 months. The rhPTH (1–34) (Shanghai Aoweihua Pharmaceutical Co., Ltd; Lot: 20061122) was subcutaneously injected once daily (20 µg) and rhPTH (1–34) treatment lasted for 18 months.

Group B: Patients received subcutaneous injection of rhPTH (1–34) (20 µg) once daily for 18 months.

Group C: Patients received PEMF therapy alone. The electromagnetic frequency was calculated based on the age, gender and BMD t value. PEMF at frequency 1 and frequency 2 was administered alternatively once every 30 s. PEMF therapy was performed for 30 min once daily for 5 months and then it was initiated 1 month later. PEMF therapy lasted for 18 months.

All the patients took calcium (600 mg) and vitamin D (125 U) once daily for 18 months.

### Detection of bone metabolism indicators and BMD

All the patients were subjected to the detection of bone turnover indicators before treatment and at 3, 6, 12, and 18 months after treatment. Blood samples were collected between 6:00 and 7:30 in the morning. Bone alkaline phosphatase (BSAP) (Immunodiagnostic System Ltd), type I procollagen N-terminal peptide (PINP) and C-terminal peptide of type I collagen (CTX/Cr) (Wampole laboraties) were measured with corresponding kits by enzyme-linked immunosorbent assay. Indirect ion electrode selection method was used for the detection of blood calcium and phosphorus. BMD was detected with Dual-Energy X-ray Absorptiometry (DEXA) instrument (Lunar, USA). The BMD (g/m^2^) of lumbar vertebrae and non-dominant femoral neck was measured.

### Statistical analysis

Data are expressed as mean ± standard deviation (SD), and statistical analysis was performed using SPSS version 19.0. Data with normal distribution were compared with one-way analysis of variance (ANOVA) among three groups. Paired t-test was used for the comparisons of data at different time points in the same group. A value of *P* < 0.05 was considered statistically significant.

## Results

### Clinical characteristics

A total of 336 PMOP patients were randomly assigned into 3 groups. There were 115 patients in the Group A, 113 in the Group B and 108 in the Group C. The mean age was 65.21 ± 7.16 years. The mean age of menopause was 20.56 ± 9.57 years. There were no significant differences in the above parameters among three groups at baseline (*P* > 0.05) (Table 1).

### BMD in different groups

**Fig. 1:**
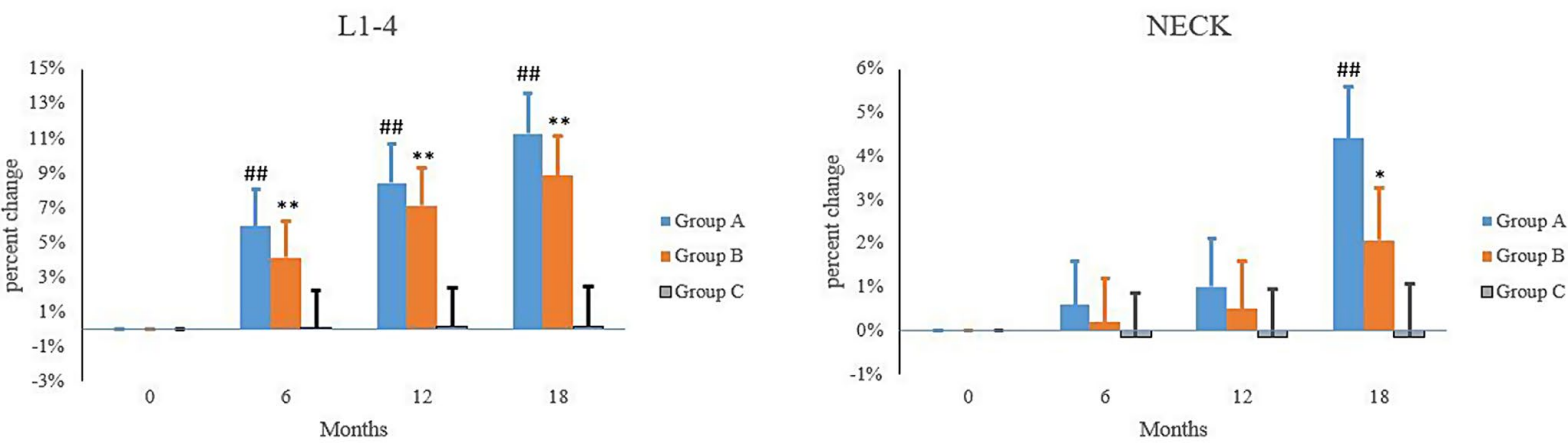
Percentage change of bone mineral density at different time points in each group. Note: Group A: ^##^*P* < 0.01 vs. before treatment; Group B: *P < 0.05, **P < 0.01 vs. before treatment

In both Group A and Group B, the lumbar spine BMD increased significantly in a time dependent manner. In the Group A, the L1-4 BMD was increased by 5.9%, 8.4% and 11.3% at 6, 12 and 18 months, respectively (*P* < 0.01 vs. before treatment); the neck BMD was increased by 1.0%, 1.2% and 4.4% at 6, 12 and 18 months, respectively, but significant difference was only noted at 18 months. In the Group B, the L1-4BMD was increased by 4.1%, 7.1% and 8.8% at 6, 12 and 18 months, respectively (*P* < 0.01 vs. before treatment); the neck BMD was increased by 1.0%, 1.0% and 2.1% at 6, 12 and 18 months, respectively, but marked difference was only noted at 18 months. In the Group C, the L1-4 BMD and Neck BMD at 6, 12 and 18 months were comparable to those before treatment (*P* > 0.05). In the Group A, the L1-4 BMD and Neck BMD were similar to those in the Group B at 6, 12 and 18 months (*P* > 0.05). The L1-4 BMD in the Group A was significantly different from that in the Group C at 6, 12 and 18 months (*P* < 0.01), but the neck BMD was comparable between groups (*P* > 0.05). In the Group B, the L1-4 BMD was significantly different from that in the Group C at 6, 12 and 18 months (*P* < 0.05, *P* < 0.01 and *P* < 0.01), but the neck BMD was comparable between groups (*P* > 0.05) (Table 2; Fig. 1).

**Table 2:** Bone mineral density at different time points in each group

Group	L1-4				Neck			
	0 month	6 months	12 months	18 months	0 months	6 months	12 months	18 months
A	0.782 ± 0.107	0.829 ± 0.113^##^	0.848 ± 0.119^##^	0.870 ± 0.127^##^	0.657 ± 0.106	0.660 ± 0.106	0.663 ± 0.107	0.678 ± 0.110^##^
B	0.786 ± 0.101	0.818 ± 0.105**	0.841 ± 0.120**	0.855 ± 0.133**	0.641 ± 0.109	0.642 ± 0.109	0.643 ± 0.109	0.658 ± 0.112*
C	0.785 ± 0.100	0.785 ± 0.100	0.786 ± 0.100	0.786 ± 0.100	0.657 ± 0.107	0.658 ± 0.107	0.658 ± 0.107	0.658 ± 0.107

Note: Group A: ^##^*P* < 0.01 vs. before treatment; Group B: *P < 0.05, **P < 0.01 vs. before treatment

### Bone turnover indicators

In both Group A and Group B, the blood calcium, BALP, PINP and CTX/Cr levels increased significantly after treatment. In the Group A, at 3, 6, 12 and 18 months, the blood calcium level was increased by 5.2%, 2.8%, 2.7% and 3.1%, respectively (*P* < 0.01 vs. before treatment); the BALP level was increased by 80.9%, 120.3%, 84.1% and 67.7%, respectively (*P* < 0.01 vs. before treatment); the PINP level was increased by 65.4%, 79.7%, 89.7% and 74.5%, respectively (*P* < 0.01 vs. before treatment); the blood CTX/Cr level was increased by 80.9%, 120.3%, 84.1% and 67.7%, respectively (*P* < 0.01 vs. before treatment).

In the Group B, at 3, 6, 12 and 18 months, the blood calcium level was increased by 5.1%, 3.3%, 3.0% and 2.1%, respectively (*P* < 0.01 vs. before treatment); the BALP level was increased by 51.6%, 81.4%, 101.1% and 56.3%, respectively (*P* < 0.01 vs. before treatment); the PINP level was increased by 48.5%, 69.8%, 80.7% and 70.5%, respectively (*P* < 0.01 vs. before treatment); the blood CTX/Cr level was increased by 29.8%, 29.9%, 55.7% and 44.8%, respectively (*P* < 0.01 vs. before treatment).

**Fig. 2:**
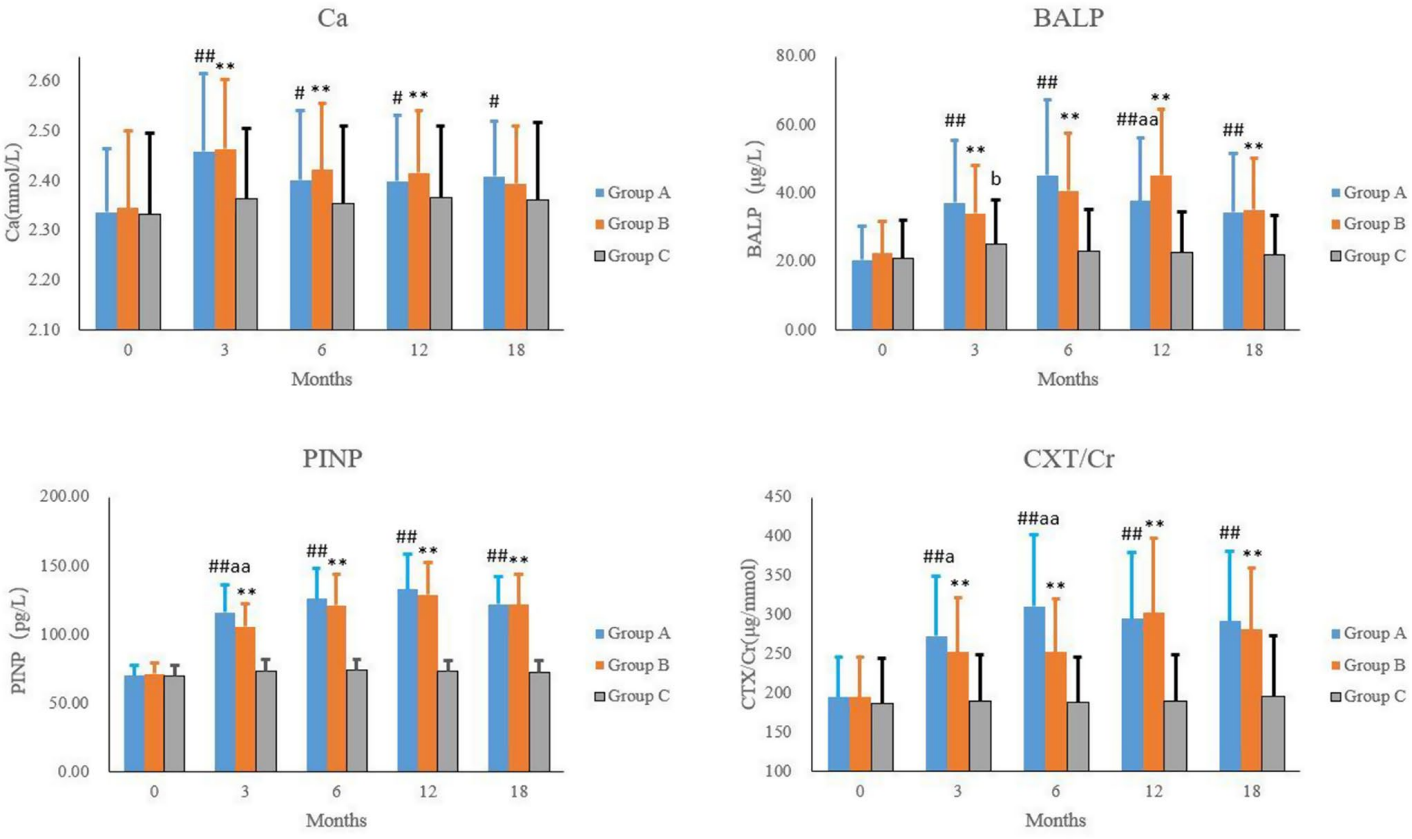
Bone turnover indicators at different time points in each group. Note: ^#^*P* < 0.05, ^##^*P* < 0.01: Group A vs. Group C; **P < 0.01: Group B vs. Group C; ^aa^*P*<0.01: Group A vs. Group B; ^b^*P*<0.05: vs. before treatment in Group C

In the Group C, the blood BALP level at 3 months was increased by 19.1% (*P* < 0.05 vs. before treatment), but the blood calcium, PINP and CTX/Cr level at all time points and the blood BALP at 6, 12 and 18 months after treatment were comparable to those before treatment (*P* > 0.05). The blood calcium, BALP, PINP and CTX/Cr at 3, 6, 12 and 18 months in both Group A and Group B were significantly different from those in the Group C (*P* < 0.01) (Fig. 2).

## Discussion

China has entered an aging society. The seventh national population census in 2021 shows that the population aged 60 years and above accounts for 18.7% (about 264 million). The prevalence of OP in the population aged 65 years and above in China is as high as 32%. Studies have revealed that OP has high incidence rate, high treatment cost, and high disability and mortality. To date, it has seriously threatened the quality of life and life expectancy of elderly people in China [4–6].

The treatment of OP mainly includes basic therapy, medication therapy, and rehabilitation therapy [1]. Among them, rhPTH (1–34) is the only osteogenesis promoting drug among the marketed drugs for treating OP, which can promote bone formation and increase BMD in patients with OP [7]. Multiple studies have shown that, as compared to other therapeutic drugs, rhPTH (1–34) can improve BMD and quality of life in OP patients more efficiently and rapidly [8]. PMOP is a systemic metabolic disorder caused by a decline in estrogen level due to the reduction in ovarian function in women. Although there is currently no complete cure for PMOP, studies have reported that rhPTH (1–34) has achieved many results in promoting osteogenesis, preventing fractures, and further improving the quality of life of PMOP patients [9]. Compared with bisphosphonates, rhPTH (1–34) can further improve femoral neck and lumbar BMD in patients and reduce the incidence of vertebral fractures [10, 11].

In as early as 1980s, PEMF was approved by the Food and Drug Administration (FDA) of United States as a commonly used physical therapy for unhealing bone fractures [12]. PEMF generates pulsed current and improves bone metabolism through stress coupling mechanism. There is evidence showing that PEMF can improve PMOP through various mechanisms, including induction of bone marrow mesenchymal stem cells (BM-MSCs), osteoblasts, osteoclasts, and osteocytes. Animal experiments have shown that PEMF can alleviate bone loss caused by estrogen deficiency by activating osteogenic progenitor cells and maintaining the osteogenic differentiation [13]. Cell experiments have indicated that PEMF may act on the BM-MSCs, osteoclasts, and osteoblasts via different mechanisms, including SIRT1 induced inhibition of NLRP3 mediated pyrolysis of BM-MSCs, regulation of osteogenic differentiation by long non-coding RNAs and related pathways, and hypoxia inducible factor driven osteoclast differentiation, which then exert anti-OP effects [14]. Clinical meta-analysis has revealed [15] that PEMF can increase improve osteogenesis and alleviate bone pain, with favorable safety [1, 16, 17]. Therefore, PEMF has become an effective physical therapy recommended by various guidelines for the diagnosis and treatment of OP in China, and PEMF has been an effective complementary therapy for PMOP.

Our previous studies [18] on PEMF have suggested that although PEMF can’t improve BMD in the short term, it can significantly improve the pain of PMOP patients and stimulate bone formation. In addition, clinical studies [19] on rhPTH (1–34) also indicate that rhPTH (1–34) can significantly increase lumbar spine BMD and improve the indicators of osteogenesis. rhPTH treatment for 18 months can also increase the BMD of femoral neck, reduce the risk of fracture at various parts, and effectively improve bone pain. Both rhPTH (1–34) and PEMF can promote bone formation, and whether the addition of rhPTH to PEMF may improve the therapeutic efficacy in PMOP patients is still poorly understood. There is evidence showing that the combination of rhPTH (1–34) and low-frequency PEMF can improve the post-operative hip joint function in the elderly patients with intertrochanteric fractures, promote the healing of fractured bone, and further reduce the recurrence of fracture [20]. Our results showed that PEMP combined with rhPTH (1–34) significantly increased the L1-4 BMD by 5.9%, 8.4%, and 11.3% at 6, 12, and 18 months, respectively, as compared to the BMD before treatment. Treatment with rhPTH (1–34) alone also markedly increased the L1-4BMD by 4.1%, 7.1%, and 8.8% at 6, 12, and 18 months, respectively, as compared to that before treatment. Although the absolute increase in the Group A was higher in the Group B, there was no significant difference between two groups. Similarly, there was no marked difference in the neck BMD between Group A and Group B. In the United States, ADA recommends that rhPTH (1–34) treatment should be shorter than 2 years. In the clinical design, the treatment with rhPTH was shorter than 2 years in the present study. More studies with large sample size are warranted to confirm our findings in the future. Studies have indicated that PEMF can improve osteogenesis. However, in the present study, the PEMP alone significantly increased BALP only at 3 months after treatment as compared to that before treatment, and the BALP, PINP, and CTX/CR (an indicator of bone resorption) at 6, 12, and 18 months were comparable to those before treatment. Significant difference in the PINP was noted between Group A and Group B only at 3 months, and there was no marked difference between groups at 6, 12, and 18 months. This might be ascribed to the study design. In our study, patients were treated with PEMF for 3 months, and then treatment was discontinued. Therefore, the indicators of bone turnover were detected at 3 months immediately after PEMF; at other time points, they were detected at the interval between two PEMF treatments. Thus, no difference was observed between two groups at other time points.

Taken together, PEMF treatment combined with rhPTH injection can significantly improve bone turnover and increase BMD in OP patients, and may become a safe and effective treatment for OP.

## Data Availability

No datasets were generated or analysed during the current study.
